# Experiences of the Built Environment, Falls and Fear of Falling Outdoors among Older Adults: An Exploratory Study and Future Directions

**DOI:** 10.3390/ijerph17041224

**Published:** 2020-02-14

**Authors:** Angela Curl, Helen Fitt, Melanie Tomintz

**Affiliations:** 1 Department of Population Health, University of Otago Christchurch, Christchurch 8013, New Zealand; 2 Centre of Excellence: Sustainable Tourism, Lincoln University, Lincoln 7647, New Zealand; helen.fitt@lincoln.ac.nz; 3 Geospatial Research Institute, University of Canterbury, Christchurch 8041, New Zealand; melanie.tomintz@canterbury.ac.nz

**Keywords:** falls, ageing, built environment, fear of falling, mobility, wellbeing

## Abstract

Falls can have serious impacts on the health, wellbeing and daily mobilities of older adults. Falls are a leading cause of injury and death amongst older adults and outdoor falls comprise a substantial proportion of pedestrian injuries. As well as physical injuries, the psychological impacts of experiencing a fall can result in older adults getting out of the house less often, resulting in lower levels of physical activity and social connection. Despite the known consequences of falls, relatively little research considers the impact of the urban built environment on falls among older adults. This research aimed to explore the experiences of older adults in the urban environment, falling and the fear of falling outdoors. We conducted an online survey with adults aged 50+ using a participatory mapping survey tool and a convenience sample. The study area was Greater Christchurch, New Zealand. Results suggest that both perceived accessibility and neighbourhood conditions are independently associated with fear of falling, after controlling for frequency of falling, gender and activities of daily living. Our findings demonstrate the need for much better understandings of the relationships between the urban environment, outdoor mobility, fear of falling and falling among older adults and we propose suggestions for future research.

## 1. Introduction 

Being mobile is important for older adults’ wellbeing. Three broad mechanisms link older adults’ mobility and wellbeing [1,2,3,4,5,6]. First, mobility facilitates access to meaningful activities and destinations outside the home. Second, movement itself is associated with both physical and mental wellbeing. Third, the potential to move, even if movement does not actually occur, is associated with wellbeing; for example, having the physical ability, financial or material resources that enable travel even if a person chooses not to travel.

Walking is an important mode of daily mobility, especially for older adults. Almost all travel involves an element of walking or wheeling, and walking contributes substantially to physical activity among an older population [7,8]. Physical activity is associated with cardio-vascular health, lower levels of obesity, diabetes, improved mood, better social connections and reduced falls risk. As a result, there is considerable policy attention being paid to the concept of active ageing, with a focus on walking as a means to achieve recommended levels of physical activity. 

The physical and psychological impacts of experiencing a fall can lead to reductions in daily mobility, decreased physical activity, social isolation and reduced confidence [9,10] among older adults, with ongoing implications for wellbeing and quality of life. 

The urban environment can impact directly on the risk of falls. Estimates of the number of falls occurring outdoors vary, but have been reported at being from around half [11,12] up to 72% [13] of all falls. The risk factors associated with outdoor falls are different to indoor falls, with outdoor fallers less likely to be frail, likely to be younger and more active [11]. As a result, the environment has a greater influence on outdoor falls than indoor falls [13,14]. Li et al. found that 73% of outdoor falls were associated with a reported environmental risk factor [13].

There is a lack of data available on the locations where outdoor falls occur and limited data on footpath conditions in many jurisdictions. This makes the identification of specific environmental features associated with outdoor falls challenging. Outdoor falls often occur as a result of a slip or trip hazard [13] and have been associated with uneven surfaces and footpath condition; wet surfaces and other poor weather conditions; footpath material; footpath obstructions; crossing the road; changing levels (e.g., stepping up or down a kerb or slopes); getting out of a vehicle; lighting; crowded places; inappropriate footwear; lack of attention or walking too fast [9,11,13,14,15,16,17]. In a longitudinal study of falls occurrence, Lee et al. [9] show that older people living in areas with more environmental barriers and worsening environmental conditions were more likely to report a fall at follow up. Greater neighbourhood walkability has been associated with higher prevalence of outdoor falls [18], although this is likely to be due to higher rates of walking, and therefore greater exposure to risk, drawing attention to the complexity of relationships between the urban environment, fear of falling and outdoor falls. 

Falls among older adults are a considerable public health issue. Around a third of adults aged over 65 fall over at least once a year [10,12,19]. In many countries, falls are the leading cause of injury resulting in hospitalisation, and in many cases, death. Longitudinal studies have shown that those who fall more frequently experience excess mortality [20]. 

Fear of falling can also have substantial impacts on older adults’ quality of life. Fear of falling is a concern about falling over which results in activity avoidance [21] and is common among older adults, whether they have experienced a fall or not [21,22]. Concerns about falling over can lead to reductions in daily mobility and associated physical activity, an increased risk of falls and threaten the wellbeing of older adults [19,23,24,25,26,27,28]. Fear of falling is associated with reduced social activity, depression, decreased quality of life, difficulties with activities of daily living and functional disability [27,28]. Individual risk factors for fear of falling have been well studied and include having experienced at least one fall and being female and older [27,29]. Although having experienced a fall is associated with fear of falling, a considerable proportion of older adults have been shown to report a fear of falling, whether they have experienced a fall or not [22] and Friedman et al. found that fear of falling is independently associated with falling and vice versa [30]. If activity is limited as a result of fear of falling, the risk of falling increases [30]. 

There is increasing evidence of relationships between the quality of the urban environment, walking behaviours, and physical and mental health and wellbeing outcomes [31,32]. Some research focuses specifically on older adults, seeking to identify environmental features that can support or discourage walking related physical activity [33]. The role of the environment in contributing to fear of falling warrants further attention. Some recent studies have focussed on environmental factors associated with fear of falling. Weather conditions such as snow, ice and wind, footpath conditions and materials, and presence of cyclists and skateboarders have been discussed in relation to fear of falling [16]. Based on self-reported environmental characteristics, Lee et al. [34] found that low traffic speeds were associated with lower odds of reporting a fear of falling. Drainage ditches and broken footpaths were associated with a greater chance of reporting a fear of falling. Building on this Lee et al. suggest that spending more time outdoors in good quality environments might reduce fear of falling amongst assisted living residents in the US [23]. 

In summary a considerable amount of research has focussed on individual risk factors for falling and fear of falling. There has also been a focus on environmental risk factors for falls occurring in the home and some attention has been paid to environmental risk factors for outdoor falls. However, there is limited research considering the relationships between urban environments, falls and fear of falling, although this is an emerging area of interest as indicated by a number of recent studies [16,23,34]. The neighbourhood experiences of older adults in relation to falls risk and fear of falling warrants further attention [16]. Beyond directly impacting a fall, is it is clear that experiences of the urban environment can play a role in fear of falling among older adults, which might be heightened among those who have experienced a fall [15]. 

In this paper we contribute to this emerging area of research through an exploration of relationships between experiences of the neighbourhood environment, falling and fear of falling in a sample of adults aged over 50 in Christchurch, New Zealand.

## 2. Materials and Methods 

### 2.1. Design

This study uses a cross-sectional survey design.

### 2.2. Participants

We used convenience and snowball sampling to recruit participants through networks of community groups working with older adults. Inclusion criteria were age 50 years or older, English speaking, residing in the Greater Christchurch (New Zealand) region, access to an electronic device to complete the questionnaire and capacity to complete an online questionnaire. Falling over and associated fear of falling can start to become a concern from quite a young age—as such we included adults over 50 in the study as we wanted to capture those who might be starting to experience concern about falling in outdoor environments. 

### 2.3. Measures

#### 2.3.1. Outcome Measures: Falls and Fear of Falling

1. Falling

We asked respondents whether they had fallen over in the last 12 months. Those who had fallen were asked a series of further questions relating to falling, including frequency of falling, location (at home, in the garden or driveway, in the street or elsewhere). Those who had fallen in the street were asked a further series of questions about the location of the fall.

2. Fear of Falling

We asked participants if they were scared of falling (yes/no). Those who reported a fear of falling were asked about their level of concern in different environments using an adapted version of the Falls Efficacy Scale (FES-I) [35]. We used seven of the 16 items from the FES-I which are specific to outdoor activities and added a further two items used by Hill et al. [36] in an expanded version of FES-I focussed on outdoor activities. The nine items were going to the shop; going up or down stairs; walking around in the neighbourhood; walking on a slippery surface; walking in a place with crowds; walking on an uneven surface; walking up or down a slope; using public transport; crossing the road. There were some missing values—five respondents did not answer all nine items, but all answered at least seven. If respondents answered at least five items we weighted their responses out of nine. Individual mean is a simple, easily interpretable and appropriate way to deal with missing data in multi-question scales [37]. Although methods such as multiple imputation may sometimes be more appropriate, it was not deemed appropriate or necessary in a study of this size. Our nine-item scale is scored from 9 to 36, with higher scores indicating a greater fear of falling. The scale has a high level of reliability (*α* = 0.93),

#### 2.3.2. Environmental Measures

1. Neighbourhood environmental conditions

We used a 12-item scale to assess difficulties with walking in the local neighbourhood. The items were based on environmental characteristics that have been associated with falls and fear of falling [15]. Respondents reported whether they experienced difficulties walking near home because of each of the following: presence/absence of footpaths; condition of footpaths; slope; width of footpath; footpath obstructions; puddles or leaves; steps or stairs; busy roads; pedestrian crossing facilities; street lighting; traffic speed; and crime. Agreement between the items is strong (*α* = 0.95). We combined all items into a single variable taking the mean of the response to the 12 items. Higher scores indicate a poorer perception of the neighbourhood environment. 

2. Perceived accessibility (PAC)

We used a self-report measure of perceived accessibility based on that developed by Lättman et al. [38]. The four item scale results in scores from 5 to 20, with higher scores indicating more positive perceptions of accessibility. Perceived accessibility is an important measure of how the accessibility of the built environment supports the needs of individuals, based on their abilities and constraints. The item has a high level of reliability (*α* = 0.93). 

#### 2.3.3. Walking

We used questions from the International Physical Activity Questionnaire-Elderly (IPAQ-E) [39] to ask about walking activity only, not other kinds of physical activity. Participants were asked on how many days they walked for at least 10 min during the last 7 days and on those days, how much time they usually spent walking. Data were cleaned, truncated and converted into categories of physical activity (PA) following the IPAQ scoring protocol. 

#### 2.3.4. Activities of Daily Living

We used a six-item scale to measure difficulties with day to day activities of daily living (ADL). Items were previously used in studies focused on outdoor mobility among mobile older adults [40,41]. Items included reporting difficulty on a scale of “not hard at all” to “too hard to do” with walking 400 m; climbing one flight of stairs; doing work around the house; doing errands such as grocery shopping; using public transport; doing moderate physical activities. There were some missing values—14 respondents did not answer all items on the ADL scale. If respondents answered at least three items we weighted their responses out of six—only one respondent answered only three items, all others answered at least four and most answered five. This scale has a range from 6 to 30, with higher scores indicating greater difficulty with daily activities and is used as a measure of functional ability. 

#### 2.3.5. Demographic

We asked respondents to report basic demographic data, including age (calculated from year of birth), gender, suburb, driver licence and living arrangements (alone, with spouse/partner, with family or other: with friends/in a retirement facility or communal home). 

### 2.4. Data Collection

Data collection took place during August and September 2018 through an online questionnaire using a map-based survey tool called Maptionnaire. A web link was distributed through the researchers’ networks of community-based groups working with older adults, including AgeConcern Canterbury, Elder Care Canterbury, the Earthquake Disability Leadership Group, Sport Canterbury, University of the Third Age (U3A), Canterbury Workers Education Association and Ōtautahi Community Housing Trust. The questionnaire took 10–15 min to complete.

### 2.5. Ethical Approval

Ethical Approval was granted by the University of Canterbury Human Ethics Committee (2017/145). A project information and privacy statement was included at the beginning of the questionnaire. Informed consent was given by completion of the questionnaire. 

### 2.6. Study Area

The study area is the Greater Christchurch urban area in New Zealand. This covers the city of Christchurch as well as the urban areas of surrounding districts: Waimakariri and Selwyn. Christchurch is the second largest city in New Zealand, with a population of 378,480 [42]. The surrounding districts of Waimakariri and Selwyn have a combined population of 120,290 [42], with an increasing urban population following the Christchurch earthquakes sequence in 2010–11, which resulted in population dispersal away from Christchurch city and into other regional centres. Both New Zealand as a whole and Christchurch have 15% of the population over 65, and 34% over 50. In Waimakariri, 19% of the population is over 65 and 40% over 50, and in Selwyn, 12% is aged over 65 and 31% over 50 [42]. 

The impacts of the earthquakes in 2010–11 on the urban environment mean that there have been considerable challenges for the pedestrian environment over recent years, which are likely to impact on experiences and perceptions of the neighbourhood environment, and footpath quality in this study [43]. The urban environment in the study area is medium to low density, with the majority of the population living in suburban environments in single low rise houses. 

### 2.7. Analyses

We describe participant characteristics for the overall sample and according to whether or not a participant reported having fallen in the past 12 months. We report associations with (a) falling and (b) fear of falling using logistic and linear regression models. We present unadjusted models for each environmental variable and covariate. We then show adjusted models for each environmental variable, controlling for each significant covariate and for sex, given the uneven sample distribution. Finally, we present a multivariate linear regression model for fear of falling, given that both environmental variables remained significant (*p* < 0.05) after controlling for covariates. All analyses were undertaken using SPSSv25. 

## 3. Results

We received 174 responses to the online survey. After cleaning the data to remove those who did not live in the Greater Christchurch region (*n* = 13) and those who did not respond to key questions (falling, or fear of falling, *n* = 32) the clean dataset contains 129 respondents. Given the low sample size in some groups, results are indicative only and should be interpreted with caution. 

### 3.1. Sample Characteristics

The characteristics of the sample are shown in Table 1. A large proportion of the sample is female (81%). The sample under-represents those in younger age groups. Those aged 50–59 constitute 13% of our sample, compared with 39% of the Greater Christchurch population aged over 50. Those aged 60–69 comprise 26% if our sample compared with 30% of the population over 50 (Statistics New Zealand, 2018). Those aged 70–79 are over-represented, comprising 48% of the sample, compared with the population (19%) whereas those aged over 80 (12%) compares with the population (11%). Table 1 also describes the characteristics of the main outcome variables: falling and fear of falling. These are discussed in the following sections. 

Most participants were reasonably active, with a high mean number of minutes walked per week. Only seven respondents reported no days walking for at least 10 min in the past 7 days and most (60%) walked for at least 10 min on five or more days in the previous week.

### 3.2. Falling

Around a third (34%) of all respondents reported having fallen over in the past 12 months. Just under half (44%) of those, or 15% of the whole sample have fallen over more than once. 

Table 1 shows that 77% of those who have fallen are female, compared with 81% of respondents being female. Interestingly those who have fallen are slightly more likely to be in the high PA category (7%) compared with the sample as a whole (4%) and to report more minutes per week walking. This may indicate that those who are more active are more exposed to the risk of falling. Those who have fallen at least once report greater difficulties with activities of daily living, poorer perceived accessibility, a greater fear of falling and poorer neighbourhood conditions than the overall sample. 

Table 2 presents logistic regression models showing odds ratios of reporting a fall in the past 12 months. Each explanatory variable is entered into a separate model. We present results for those who have fallen at least once in the previous year compared with those who have not fallen. The first column shows unadjusted bivariate associations. The second column shows associations with the environmental variables adjusted for activities of daily living and gender. Both neighbourhood conditions and perceived accessibility show significant associations with having fallen. Those with poorer perceptions of their neighbourhood environment are more likely to have fallen (OR: 1.51). Those who have better perceived accessibility are less likely to report having fallen (OR: 0.87). However, after adjusting for activities of daily living and gender these associations are not significant. 

#### Location of Falls

We also asked respondents who reported having fallen (*n* = 44) where their fall (s) occurred. The responses were as follows, shown as a percentage of those who reported a fall:

- Fallen at home: 35%
- Fallen in the garden or driveway: 35%
- Fallen in the street: 30%
- Fallen elsewhere: 32%

While 10% of the entire sample reported having fallen in the street, given the small sample size it is difficult to undertake any further analyses specific to those who have fallen outdoors. 

Of those who reported falling elsewhere, free text responses suggest locations such as public buildings (libraries, hospitals, restaurants, churches), open spaces (parks, back country, wetlands, car parks) and while getting in/out of vehicles. 

### 3.3. Fear of Falling

This section reports associations with fear of falling. We report associations with both the binary outcome variable for fear of falling (Table 3) and the adapted fear of falling scale (Table 4).

Overall, 43% of respondents reported a fear of falling. To some extent this is skewed by females being over-represented in our sample, given they are more likely to report a fear of falling: 50% of females, compared with 17% of males report a fear of falling.

A total of 52% of those who have fallen, report a fear of falling, compared to 38% of those who have not fallen. Although there is no significant association between having fallen at all and fear of falling, those who have fallen more than once are more likely to report a fear of falling (OR: 2.59). 

Those who report a fear of falling also report greater difficulties with activities of daily living (OR: 1.21), poorer perceptions of accessibility (OR: 0.79), and poorer neighbourhood conditions (OR: 1.50).

Perceived accessibility is significantly associated with fear of falling (OR: 0.78), after adjusting for ADL and gender, but neighbourhood conditions are not (OR: 1.27). 

Table 4 shows the results of linear regression models with the fear of falling scale as the outcome variable. Similarly to the binary outcome fear of falling, the fear of falling scale scores are greater for those who report poorer neighbourhood conditions (2.10), poorer perceived accessibility (−0.81), more difficulties with activities of daily living (0.81), those who have fallen more than once (5.53) and lower for males (−3.92). 

Given that both environmental variables remained significant after controlling for ADL, falling and gender, we also tested a full model with both environmental variables included in the same model, as opposed to separate models in Table 4. As shown in Table 5 both perceived accessibility (−0.47) and neighbourhood conditions (1.15) are independently associated with fear of falling, after controlling for frequency of falling, gender and activities of daily living.

## 4. Discussion

Our results indicate that poor neighbourhood conditions and poor perceived accessibility are associated with a fear of falling among survey respondents. 

Fear of falling among our respondents is greater for those who report poorer neighbourhood conditions. This is consistent with other recent research, which has shown that those who fear falling have poorer perceptions of the physical environment [22,34]. Our measure of neighbourhood conditions included items related to urban environments that have been found to be related to falling and a fear of falling in previous research. We cannot ascertain causality in this study, but the strong association between poorer perceptions of the neighbourhood environment and fear of falling should be a cause for concern. Those who experience a fear of falling avoid activities such as going outdoors [21]. Even if the environmental conditions have not caused a fear of falling, the results of this study demonstrate the importance of environmental conditions to those who fear falling. That neighbourhood conditions are associated with increased anxiety related to falling, which itself can result in poorer wellbeing and quality of life, has parallels with other research which has suggested that walking outdoors in poor urban environments might not promote wellbeing [44]. Therefore, efforts to promote walking as part of healthy active ageing agendas should consider the environment in which this takes place, as in some cases promoting walking in poor environments could have adverse effects such as fear of falling.

Previous research has noted heightened environmental awareness following a fall [15] which suggests there could be some value in future research investigating the moderating effect of having fallen on relationships between neighbourhood conditions and fear of falling. Falls on footpaths and streets are more likely to result in injury than recreational walking, particularly in more deprived areas [12] where neighbourhood conditions are often poorer, suggesting that this fear of falling related to neighbourhood conditions is not unwarranted. However, in this study the relationship between neighbourhood conditions and having fallen in the past year did not hold in the adjusted model. 

Similarly, poorer perceived accessibility is associated with fear of falling, but not falling in the adjusted model. Given that fear of falling has been described as an “exaggerated concern” [23] it follows that those who are concerned about falling might have deflated beliefs regarding their level of accessibility in the local neighbourhood. Where this perception of accessibility influences behaviour then the range of destinations and social connections available to those with a fear of falling are compressed. 

Fear of falling was more prevalent (44%) than having fallen over (34%), estimates which are broadly consistent with other research. Those who had fallen more than once were almost three times as likely to report a fear of falling (Table 4). However, a considerable number of participants (25%) expressed concern about falling, despite not having fallen over in the past year. Others have attempted to understand ‘which comes first’: falls, or fear of falling [30], concluding that each is a risk factor for the other whereby developing a fear of falling or experiencing a fall can lead to a spiralling falls risk, concern about falling and declines in mobility. The strong associations between neighbourhood conditions, perceived accessibility and fear of falling warrants further research understanding the role of the urban environment in contributing to development of a fear of falling, and the potential of good quality environments to mitigate against this. 

Interactions with individual risk factors should also be considered. For example, the results of this study demonstrate that males are considerably less likely than females to report a fear of falling. Previous research supports this [27,29,34] and has found that females are more likely to fear moving outdoors [45]. However, studies tend to treat sex as a covariate and has not explored the mechanisms that might explain such disparities, beyond a recognition that females have a higher prevalence of osteoporosis and musculoskeletal conditions [28]. Despite evidence of an enhanced fear of falling, females do not fall over outdoors any more frequently than males [46], potentially because they restrict outdoor walking as a result of concerns about falling over. Future research should consider in more depth the role of gender in fear of falling and the implications for risk of falls, outdoor mobility and quality of life as well as interactions with urban environments. 

Associations with walking related physical activity were not significant in this study. This is potentially due to the complex relationships between outdoor mobility, falling and fear of falling. The direction of coefficients in our results suggest that higher levels of walking are associated with a lower likelihood of a fear of falling but higher odds of having experienced a fall. Other research has found that high leisure time physical activity is associated with increased risk of outdoor, but not indoor falls [13] because of increased exposure to risk [12]. Given the most common behaviour associated with outdoor falls is walking [46], it is unsurprising that those who walk more experience more falls. Given the small sample size, we did not distinguish between indoor and outdoor falls in our analyses, which may explain our non-significant findings. Other studies have compared fear of falling among those who do and do not go outdoors independently and found significant associations, among assisted living residents [23]. However, in our sample most respondents (95.3%) had walked outside for at least 10 minutes on at least one day in the previous week. It is perhaps, then, surprising that we find such high rates of concern about falling, in a relatively young and active sample. 

Despite a broad age range of respondents (51–87), there were no associations of age with falling or fear of falling. This demonstrates the importance of considering functional ability (measured using ADL) rather than biological age and supports extending studies on this topic to consider the younger-old. Injury data [47] shows an increased prevalence of falls in the road or street with age, with substantial increases for those over 60. Although the risk of injury may increase with age, the broader impacts of (potentially non-injurious) falls at a younger age on fear of falling and confidence going outdoors should be given more consideration. Especially when focussing on outdoor falls, those who are experiencing reduced functional abilities may start to fall outside from a much younger age than is typically included in studies of ‘older’ adults. In fact, 16% of those who had fallen over were in the 50–59 age group, slightly higher than the population (13%) in that age range—although this is likely because of the convenience sample which would appeal to those for whom the topic is pertinent. 

### 4.1. Limitations

We used a convenience sample, limiting the representativeness of the sample and so the population inferences that can be made based on these results. However, the prevalence of falls mirrors that found in many other studies indicating that around a third of older adults have fallen over in the past year [10,12,19]. Given that only around a third of these falls happened in the street (*n* = 13), we could not undertake any analyses that focussed on falls in the street. This prevalence of falls in the street can usefully inform sample size calculations for future population surveys focussed on outdoor falls. 

Use of online self-completion questionnaires can be problematic, especially amongst our study population. This was the only option for this exploratory study, but future studies should utilise other questionnaire formats. In particular, the online completion means we do not know how well individuals were able to engage and we may have excluded some participants as a result.

The relationships between urban environments, outdoor mobility, falling and fear of falling are complex and intertwined. Our cross-sectional study is limited in its ability to make causal inferences. There is scope for future research to consider the role of the urban environment in mitigating against fear of falling. 

We have focussed on aspects of the physical environment, but the social environment is known to be important for outdoor walking [48], something we did not account for in this study, beyond living arrangements (alone/with others). The social environment is also important in fear of falling and perceptions of falls risk [17] and should be considered in future research.

### 4.2. Future Research Directions

This is an exploratory study, which adds to the limited literature on relationships between the urban environment, outdoor mobility, fear of falling and falling among older adults. A particular contribution of this study is to highlight a number of areas for future research. We suggest that there is a need to focus more on the complexity and reciprocal nature of relationships between urban environments, outdoor mobility, falls, fear of falls and falls risk. This might require more complex modelling of causal pathways using structural equation modelling, for example. In order to do this, future surveys need sample sizes sufficient to be able to analyse outdoor falls specifically. In our questionnaire, we asked a series of questions specific to those who had fallen in the street, including familiarity with the area, social support, causes of the fall and reporting of falls, but despite a reasonably high prevalence of falls in the street, the sample size limited further analysis. These attributes of outdoor falls can provide a focus for future research. 

Drawing together the points raised in the discussion, we outline below some future research directions and propose a conceptual model for understanding relationships between urban environments, outdoor mobility, falling and fear of falling outdoors.

**Figure 1:**
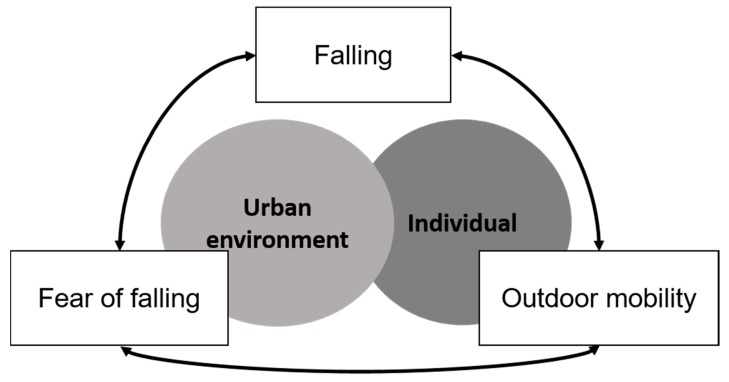
A proposed conceptual framework for exploring the relationships between fear of falling, falling and outdoor mobility, and the role of environmental and individual risk factors.

- Is there a moderating effect of having experienced a fall on relationships between urban environments and fear of falling?
- What contribution do neighbourhood conditions to developing a fear of falling?
- How do neighbourhood conditions and individual risk factors interact to influence falling and fear of falling?
- How does the social and environmental context of falls influence outdoor falls?
- What is the relationship between the urban environment, outdoor mobility, falls and fear of falling in deprived areas, where neighbourhood conditions are likely to be poorer and rates of walking higher?
- Does new evidence support the conceptualisation of reciprocal and complex relationships between falling, fear of falling and outdoor mobility, and the contribution of urban environments and individual risk factors (Figure 1)

## 5. Conclusions

This study contributes to an emerging literature considering the urban environment as a risk factor for falls and fear of falling. We found associations between two measures of the neighbourhood environment: neighbourhood conditions and perceived accessibility, and a fear of falling over outdoors. Relationships between the neighbourhood environment and falling did not hold after adjusting for covariates, but our measure was not specific to outdoor falls. 

Falls are a considerable public health concern from an injury perspective, but more attention needs to be paid to the ongoing implications of falls for outdoor mobility, fear of falling and experiences of the urban environment. Furthermore, the implications of fear of falling, regardless of having experienced a fall, in relation to the urban environment demand more attention, given the implications of fear of falling for outdoor mobility and quality of life. Understanding the perspectives and experiences of the urban environment among those who have fallen or fear falling can be important for prevention programmes [49]. 

Falls, fear of falling and moderation of daily activities to avoid potential falls can all negatively influence the wellbeing of older adults. They do so by limiting meaningful activities outside the home, by compromising the enjoyment (and other physical and mental benefits) of movement itself, and by restricting people’s own sense of their potential to move. A better understanding of the complex relationships between the different variables explored in this paper could facilitate the development of effective programmes to help people maintain quality of life into later older age. Relevant programmes could include infrastructural works, urban planning protocols, physical conditioning and exercise programmes and psychological tools and techniques to help with fear management. Currently, however, it is difficult to determine an appropriate mix of such programmes. In the context of globally ageing populations, improving understandings of this topic could increase the efficacy of government spending as well as improving quality of life for ageing populations around the world. 

## Figures and Tables

**Table 1:** Sample characteristics (column%).

Variable	Total Sample *n* = 129 * (%)	Fallen *n* = 44 (%)	Fear of Falling *n* = 55 (%)
Gender: Female	99 (81)	77	93
Age 50–59	17 (13)	16	15
Age 60–69	34 (26)	34	26
Age 70–79	62 (48)	39	46
Age 80+	16 (12)	11	15
Driver licence	121 (95)	96	93
Lives alone	38 (30)	34	32
Live with spouse/family	88 (68)	66	69
Walking related PA: Low	58 (50)	55	54
Walking related PA: Medium	53 (46)	38	42
Walking related PA: High	5 (4)	7	4
	**Sample: Mean (sd)**	**Fallen: Mean (sd)**	**Fear of Falling: Mean (sd)**
Age	70.5 (7.9)	70.1 (8.1)	70.6 (8.1)
Activities of daily diving (scale)	6.3 (3.5)	8.1 (4.1)	8.1 (3.9)
Perceived accessibility	17.3 (3.5)	16.1 (3.8)	15.8 (3.9)
Minutes walked per week	315 (295)	336 (359)	276 (295)
Neighbourhood conditions	2.3 (1.1)	2.6 (1.1)	2.5 (1.2)
Fear of falling (scale)	13.2 (61)	15.0 (7.4)	-

* Numbers may not add to 129 where there are missing responses. Percentages may not add to 100 due to rounding.

**Table 2:** Logistic regression models—odds ratios (OR) demonstrate the odds of having fallen in the past 12 months for each key variable.

Covariate	Fallen at Least Once (Unadjusted) OR (CI)	Fallen at Least Once (Adjusted for ADL and Gender) OR (CI)
Neighbourhood conditions	NR^2^ 0.06 1.51 (1.06, 2.16) *	NR^2^ 0.11 0.35 (0.98, 2.04)
Perceived accessibility	NR^2^ 0.07 0.87 (0.78, 0.97)*	NR^2^ 0.07 0.94 (0.80, 1.04)
Walking related PA (ref: Low)	NR^2^ 0.03	
Walking related PA: Medium	0.66 (0.30, 1.15)	
Walking related PA: High	2.28 (0.35, 14.74)	
Mins per week walking	NR^2^ 0.004 1.00 (0.99, 1.002)	
Fear falling (ref: no fear)	NR^2^ 0.03 1.81 (0.87, 3.79)	
Fear of falling scale	NR^2^ 0.06 1.07 (1.01, 1.14)	
Age	NR^2^ 0.02 0.99 (0.95, 1.04)	
Male (ref: female)	NR^2^ 0.06 1.43 (0.57, 3.56)	
Live alone (ref: living with others)	NR^2^ 0.01 1.37 (0.63, 3.01)	
Driver licence (ref: no licence)	NR^2^ 0.001 1.33 (0.25, 7.15)	
Activities of daily living	NR^2^ 0.07 1.15 (1.03, 1.29) *	

1 NR^2^—Nagelkerke R-squared measure of model fit. 2 * indicates significance at *p* < 0.05.

**Table 3:** Logistic regression models—odds ratios (OR) demonstrate the odds of having reported a fear of falling for each key variable.

Covariate	Fear of Falling (Unadjusted) OR (CI)	Fear of Falling (Adjusted for Falling, ADL and Gender) OR (CI)
Neighbourhood conditions	NR^2^ 0.06 1.50 (1.06, 2.12) *	NR^2^ 0.27 1.27 (0.87, 1.86)
Perceived accessibility (PAC)	NR^2^ 0.18 0.79 (0.70, 0.89) **	NR^2^ 0.33 0.78 (0.66, 0.93) **
Walking related PA (ref: Low)	NR^2^ 0.07	
Walking related PA: Medium	0.75 (0.35, 1.59)	
Walking related PA: High	0.82 (0.13, 5.28)	
Mins per week walking	NR^2^ 0.02 0.99 (0.99, 1.00)	
Frequency of falling (ref: not fallen)	NR^2^ 0.06	
Fallen once	1.1 (0.44, 2.75)	
Fallen more than once	2.59 (1.24, 10.38) *	
Age	NR^2^ 0.00 1.00 (0.96,1.05)	
Male (ref: female)	NR^2^ 0.10 0.20 (0.06, 0.62) *	
Live alone (ref: living with others)	NR^2^ 0.02 1.16 (0.54, 2.49)	
Driver licence (ref: no licence)	NR^2^ 0.07 0.53 (0.11, 2.46)	
Activities of daily living (ADL)	NR^2^ 0.11 1.21 (1.06, 1.38) *	

1 NR^2^—Nagelkerke R-squared measure of model fit. 2 * indicates significance at *p* < 0.05, ** at *p* < 0.01.

**Table 4:** Linear regression models—associations with fear of falling scale.

Covariate	Fear of Falling Scale (Unadjusted)	Fear of Falling Scale (Adjusted for ADL, Falling and Gender)
Neighbourhood conditions	2.10 (1.56, 3.05) ** R^2^ 0.14	1.38 (0.54, 2.22) ** R^2^ 0.41
Perceived accessibility (PAC)	−0.81 (−1.08, −0.55) ** R^2^ 0.23	−0.55 (−0.85, −0.47) ** R^2^ 0.41
Walking related PA (ref: Low)	R^2^ 0.03	
Walking related PA: Medium	−1.96 (−4.42, 0.33)	
Walking related PA: High	−2.97 (−8.57, 2.63)	
Mins per week walking	−0.004 (−0.008, −0.001) R^2^ 0.05	
Fallen once	0.51 (−2.10, 3.11)	
Fallen more than once	5.53 (2.63, 8.43) ** R^2^ 0.10	
Age	0.08 (−0.05, 0.21) R^2^ 0.01	
Male	−3.92 (−657, −1.26) ** R^2^ 0.07	
Live alone	2.11 (−0.18, 4.41) R^2^ 0.03	
Driver licence	−1.78 (−6.46, 2.90) R^2^ 0.04	
Activities of daily living (ADL)	0.81 (0.54, 1.09) ** R^2^ 0.21	

1 ** indicates significance at *p* < 0.01.

**Table 5:** Linear regression model for fear of falling.

Covariate	Fear of Falling Scale
Neighbourhood conditions	1.15 (0.32, 1.99) **
Perceived accessibility (PAC)	−0.47 (−0.78, −0.16) **
Fallen once	−0.13 (−2.42, 2.15)
Fallen more than once	2.98 (0.41, 5.55) *
Male	−4.12 (−6.44, −1.80) **
Activities of daily living (ADL)	0.51 (0.19, 0.83) **
R^2^ 0.42	

1* indicates significance at *p* < 0.05, ** at *p* < 0.01.
